# Advanced glycation endproducts link inflammatory cues to upregulation of galectin-1 in diabetic retinopathy

**DOI:** 10.1038/s41598-017-16499-8

**Published:** 2017-11-23

**Authors:** Atsuhiro Kanda, Yoko Dong, Kousuke Noda, Wataru Saito, Susumu Ishida

**Affiliations:** 1 Laboratory of Ocular Cell Biology and Visual Science, Department of Ophthalmology, Faculty of Medicine and Graduate School of Medicine, Hokkaido University, Sapporo, Hokkaido, 060-8638 Japan; 2 Department of Ophthalmology, Faculty of Medicine and Graduate School of Medicine, Hokkaido University, Sapporo, Hokkaido, 060-8638 Japan

## Abstract

Diabetic retinopathy (DR) is an inflammatory and progressive vaso-occlusive disease resulting in angiogenesis. Galectin-1 is a hypoxia-induced angiogenic factor associated with cancer and proliferative DR. Here we reveal a significant upregulation of galectin-1 in eyes of DR patients along with progression of clinical stages beginning from the pre-ischemic, inflammatory stage with diabetic macular edema, but not in eyes with non-diabetic retinal vascular occlusions. As for its regulatory mechanism unrelated to hypoxia but selective to DR, *in vitro* galectin-1/*LGALS1* expression was shown to increase after application to Müller glial cells with interleukin (IL)-1β, which was induced in monocyte-derived macrophages and microglial cells via toll-like receptor (TLR) 4 signaling stimulated by advanced glycation endproducts (AGE). *In vivo* inhibition of AGE generation with aminoguanidine, macrophage depletion with clodronate liposomes, and antibody-based blockade of Il-1β and Tlr4 attenuated diabetes-induced retinal *Lgals1* expression in mice. Fibrovascular tissues from proliferative DR eyes were immunoreactive for AGE, TRL4 and IL-1β in macrophages, and IL-1β receptor-positive glial cells expressed galectin-1. Therefore, diabetes-induced retinal AGE accumulation was suggested to activate IL-1β-related inflammatory cues in macrophages followed by Müller cells, linking to galectin-1 upregulation in human DR with time. Our data highlight AGE-triggered inflammation as the DR-selective inducer of galectin-1.

## Introduction

Diabetic retinopathy (DR) is the most common microvascular complication in patients with diabetes, and may have a debilitating impact on visual acuity, eventually leading to blindness. Diabetic macular edema (DME), involving retinal thickening in the macular area, occurs after breakdown of the blood-retinal barrier characterized by inflammatory leakage from dilated hyperpermeable capillaries and microaneurysms. Subsequently, microvascular occlusion or regression (*i.e*., capillary dropout), a characteristic feature seen in progressive DR, initiates ischemia-induced neovascularization^1^. The advanced stages of DR, proliferative DR (PDR) followed by neovascular glaucoma (NVG), develop fibrovascular proliferation whereby abnormal new blood vessels and fibrous tissues grow on the surface of the retina, iris and angle, resulting in severe complications including vitreous hemorrhage, traction retinal detachment and intraocular pressure rise.

Studies have shown that the pathogenesis of DR involves activation of leukocytes (*e.g*., monocytes, granulocytes and lymphocytes), sharing similarities with chronic inflammatory diseases^1,2^. Activated leukocytes secrete many growth factors and cytokines including vascular endothelial growth factor (VEGF), interleukin (IL)-1β, and interferon (IFN)-γ, and cause retinal microvascular injury and leakage, suggesting that inflammation plays a significant role in the onset and progression of DR, especially at the pre-ischemic stage of DME^3–7^. Among the various biological pathways involved in diabetic complications, the generation and accumulation of advanced glycation endproducts (AGE), a complex and heterogeneous group of biochemical compounds that are formed through nonenzymatic glycation of proteins, lipids and nucleic acids, have been considered as one of the major initiators for inflammatory response in diabetes^8–10^. The current management for DR requires optimal glycemic control to slow the disease progression, and the intravitreal injection of VEGF blockers is a first-line treatment for DME; however, the anti-VEGF strategy is not necessarily effective for all patients with DME, suggesting the potential involvement of other molecules in the pathogenesis of DR.

Galectins, an evolutionarily conserved family of galactoside-binding lectin proteins, bind to cell surface glycol-conjugated proteins or lipids, and regulate a myriad of biological reactions without having specific receptors like cytokines do^11^. Galectin-1, encoded by the *LGALS1* gene, contributes to cell adhesion/proliferation and immunosuppression in a variety of cancer cells and regulatory T lymphocytes, respectively^12,13^. Recently, we and others have revealed that galectin-1 interacts with the *N*-glycans of VEGFR2, enhancing phosphorylation of VEGFR2 and activating its downstream signal transduction in endothelial cells, so as to promote hypoxia-induced angiogenesis^14,15^, together with VEGFR1-mediated vascular hyperpermeability^16^. Importantly, vitreous aspirates from eyes with PDR showed higher protein levels of galectin-1 than those from non-diabetic controls^15^. Moreover, the elevated levels of galectin-1 were not correlated with VEGF levels also increased in PDR eyes, suggesting that these two pro-angiogenic molecules are independently regulated^15^.

In this study, we investigated protein levels of galectin-1 in eyes with the different clinical stages of DR, and explored upstream regulatory stimuli for hypoxia-unrelated galectin-1 expression selectively in the pathogenesis of DR but not non-diabetic retinal vascular occlusions.

## Results

### Elevation of Galectin-1 Protein Levels Selectively in Eyes with DR

**Figure 1:**
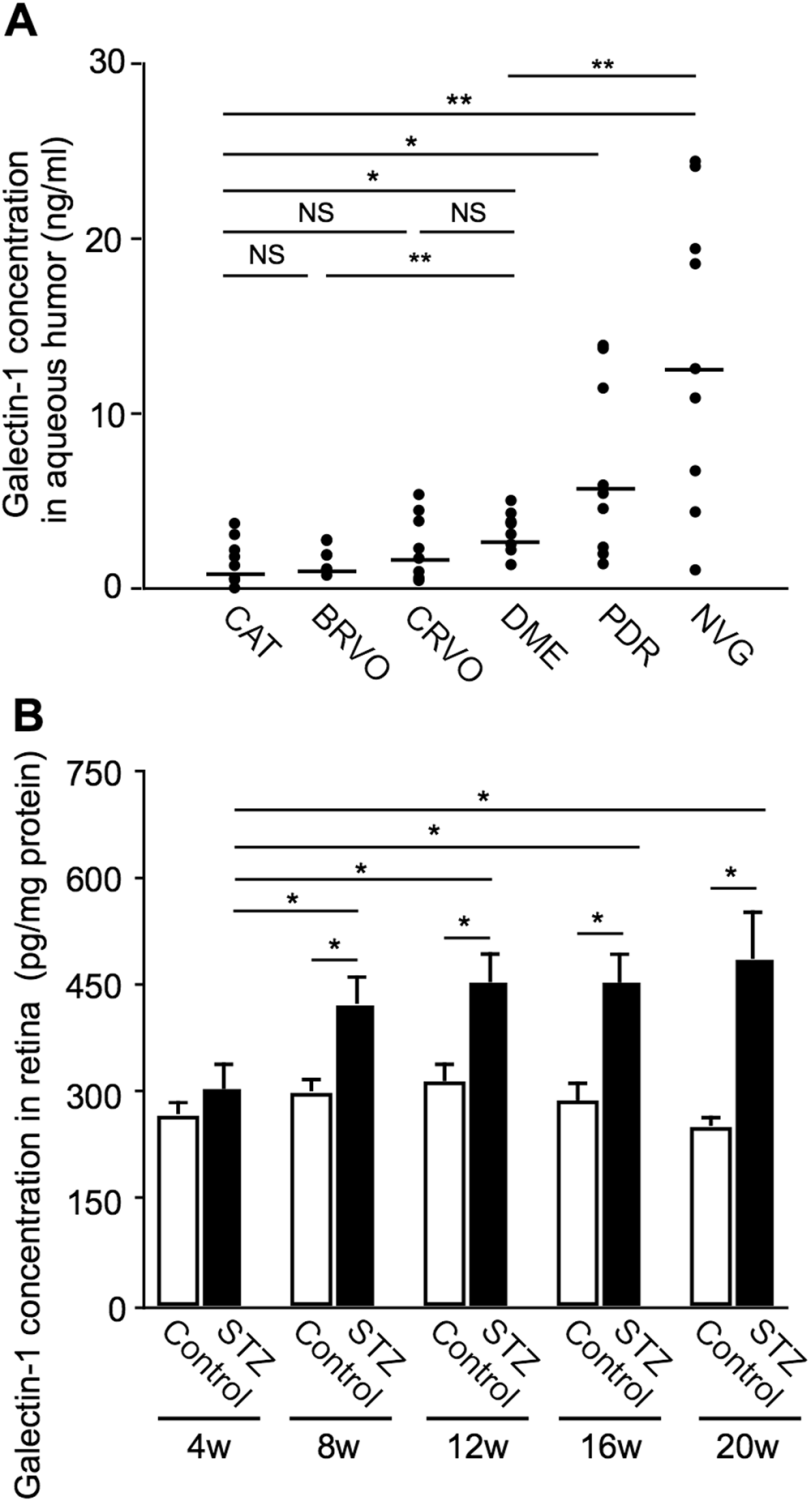
Elevation of galectin-1 protein levels selectively in eyes with DR. (**A**) Protein levels of galectin-1 in eyes with CAT (n = 8), DME (n = 7), PDR (n = 9), NVG (n = 9), BRVO (n = 7), and CRVO (n = 10). Black symbols indicate individual samples in each group with a bar showing the average. (**B**) Galectin-1 protein expression in retinal tissues from mice with STZ-induced diabetes (n = 6 per group). *p < 0.05, **p < 0.01.

We previously reported that galectin-1 levels significantly increased in the vitreous fluid of PDR eyes compared with non-diabetic controls (idiopathic macular hole and epiretinal membrane)^15^. To further investigate the involvement of galectin-1 in the worsening course of DR, we performed enzyme-linked immunosorbent assay (ELISA) experiments to measure galectin-1 protein levels in aqueous humor samples collected from DR eyes with different clinical stages of DME, PDR, and NVG and from control eyes of non-diabetic, age-matched subjects with cataract (CAT) alone. Galectin-1 protein levels in eyes with DME (3.23 ± 0.42 ng/ml), PDR (6.74 ± 1.67 ng/ml) and NVG (13.58 ± 2.59 ng/ml) were significantly higher than those with CAT (1.63 ± 0.46 ng/ml) (Fig. 1A). Galectin-1 protein levels showed a soaring rise with the progression of clinical stages of DR, in consistence with a recent report with PDR and NVG^17^. However, ours is the first to show the elevated levels of galectin-1 in eyes with DME, a hallmark of diabetic microvascular inflammation at the pre-ischemic stage prior to the angiogenic stages of PDR and NVG. In addition, we evaluated galectin-1 protein levels in aqueous humor samples collected from eyes with macular edema due to branch retinal vein occlusion (BRVO) and central retinal vein occlusion (CRVO). Interestingly, these non-diabetic retinal vaso-occlusive diseases showed no significant differences in galectin-1 protein levels compared with CAT (BRVO, 1.64 ± 0.28 ng/ml; CRVO, 2.44 ± 0.67 ng/ml) (Fig. 1A), suggesting a potential selectivity for DR in the ischemia-independent regulation of galectin-1. Indeed, the mean galectin-1 levels were still higher in eyes with DME than in those with retinal vein occlusions, showing a statistically significant difference compared to BRVO and a nonsignificant tendency to CRVO (Fig. 1A).

Consistent with our current and previous results using clinical samples, galectin-1 protein levels gradually increased in the retina of mice with streptozotocin (STZ)-induced diabetes over time (303.7 ± 37.5, 423.7 ± 38.4, 453.8 ± 44.0, 452.4 ± 41.6, and 488.7 ± 67.2 pg/mg at 4, 8, 12, 16, and 20 weeks after STZ injection, respectively) compared to citrate buffer as a vehicle control (269.3 ± 16.7, 301.4 ± 20.9, 315.5 ± 27.9, 288.5 ± 27.8, and 252.9 ± 16.7 pg/mg at 4, 8, 12, 16, and 20 weeks, respectively) (Fig. 1B).

### Induction of *LGALS1* mRNA Exclusively in IL-1β-Stimulated Müller Glial Cells

**Figure 2:**
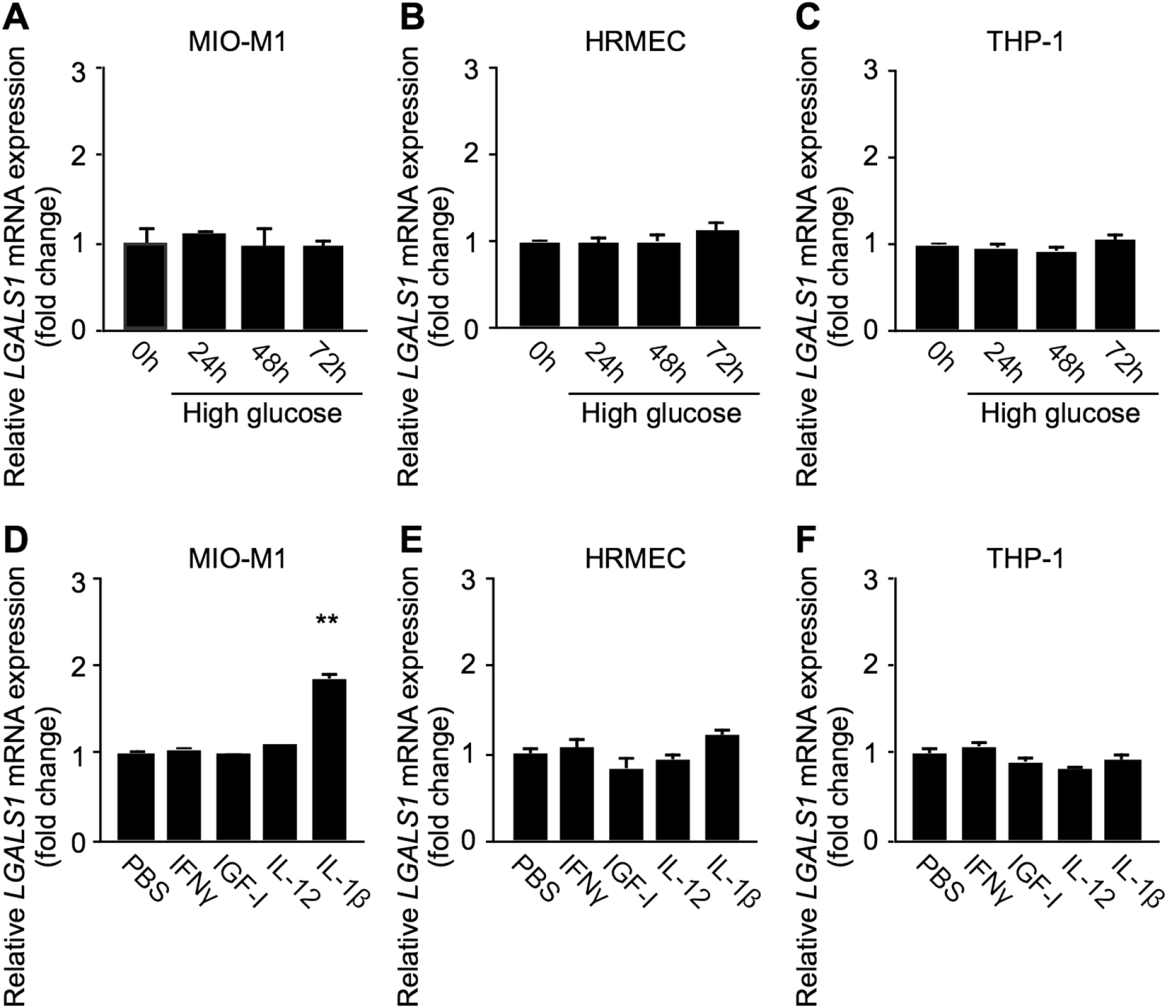
Induction of *LGALS1* mRNA exclusively in IL-1β-stimulated Müller glial cells. (**A–C**) MIO-M1 (**A**), HRMEC (**B**), and THP-1 (**C**) were incubated with the medium containing 30-mM glucose for indicated time intervals, and *LGALS1* gene expression levels were analyzed. (**D–F**) MIO-M1 (**D**), HRMEC (**E**), and THP-1 (**F**) were treated with IFN-γ (100 ng/ml), IGF-I (100 ng/ml), IL-12 (10 ng/ml), and IL-1β (10 ng/ml) for 24 hours, and *LGALS1* expression was analyzed. **p < 0.01 (n = 8 per group).

Hypoxia-inducible factor (HIF)-1α, a master transcription factor for cellular response to hypoxia, was shown to regulate *LGALS1* mRNA expression in cancer cells^13^. Similarly, we recently demonstrated a significant induction of *LGALS1* mRNA expression following hypoxic insults to various retinal cells^15^. More recently, ischemia-induced retinal neovascularization in mice was shown to overexpress galectin-1^18^. However, the unelevated galectin-1 levels in eyes with BRVO and CRVO (Fig. 1A), two representative vaso-occlusive diseases characterized by retinal ischemia^19^, led us to hypothesize a DR-selective regulatory mechanism other than hypoxia. First, we checked high glucose application to 3 major cell types closely associated with the pathogenesis of DR: Müller glial cells (MIO-M1), retinal microvascular endothelial cells (HRMEC), and monocyte-derived macrophages (THP-1). *LGALS1* mRNA levels were unaltered in these cells during culture with 30-mM glucose up to 72 hours in comparison to osmolality-controlled 5-mM glucose at 0 hour (Fig. 2A–C).

Next, we extracted the following 4 inflammatory cytokines from the literature^3–7^: IFN-γ, insulin-like growth factor (IGF)-I, IL-12, and IL-1β; all of which were shown to increase exclusively in eyes with DR but not BRVO or CRVO, as candidate inducers for hypoxia-independent *LGALS1* gene expression in these 3 cell lines. Importantly, *LGALS1* mRNA levels significantly increased solely after IL-1β stimulation to MIO-M1 (fold change = 1.86) compared to PBS as a vehicle control, but not after any of the other combinations of cytokines and cell types (Fig. 2D–F).

Given that Müller glial cells were reported to express VEGF and galectin-1 in fibrovascular tissues in human PDR^15,20^, we examined the *in vitro* induction of these two angiogenic factors under hypoxic and IL-1β stimuli to MIO-M1 (Supplementary Fig. S1). *LGALS1* and *VEGF165* mRNA levels significantly increased at 6 hours after treatment with IL-1β and hypoxia, respectively. At 24 hours, both molecules were upregulated mainly following either of these stimuli, still showing the preferential induction of *LGALS1* by IL-1β and *VEGF165* by hypoxia.

### Galectin-1/*LGALS1* Expression in Müller Glial Cells via IL-1β-IL1R1 Signaling

**Figure 3:**
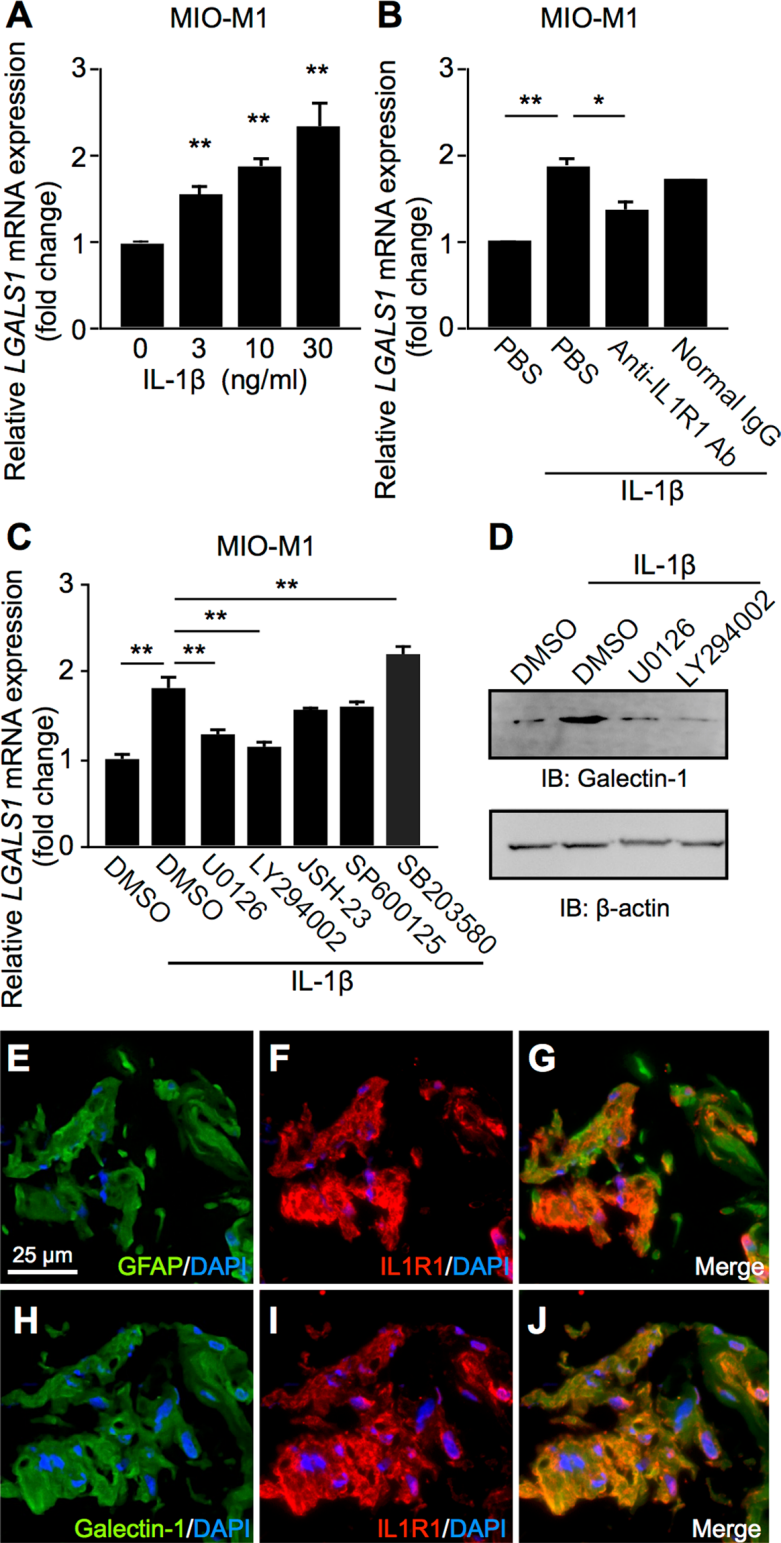
Galectin-1/*LGALS1* expression in Müller glial cells via IL-1β-IL1R1 signaling. (**A**) MIO-M1 was treated with IL-1β (3–30 ng/ml) for 24 hours, and *LGALS1* expression was analyzed. (**B**) MIO-M1 was pretreated with a neutralizing antibody against IL1R1 (10 μg/ml) and control normal IgG (10 μg/ml) for 30 minutes followed by treatment with IL-1β (10 ng/ml) for 24 hours, and *LGALS1* gene expression levels were analyzed. (**C–D**) MIO-M1 was pretreated with each inhibitor at 10 μM for 30 minutes before cultured with IL-1β (10 ng/ml) for 24 hours, and *LGALS1* gene and protein expression levels were analyzed. *p < 0.05, **p < 0.01 (n = 8 per group). Full-length blots are presented in Supplementary Fig. S3. (**E–G**) Double labeling of GFAP (*green*), IL1R1 (*red*) and DAPI (*blue*) in PDR fibrovascular tissues. (**H–J**) Double labeling of galectin-1 (*green*), IL1R1 (*red*) and DAPI (*blue*) in PDR fibrovascular tissues. Scale bar = 25 μm.

To confirm IL-1β-induced *LGALS1* gene expression in Müller glial cells (Fig. 2D), we performed additional experiments. IL-1β application to MIO-M1 elevated *LGALS1* mRNA levels in a dose-dependent manner (3 ng/ml, fold change = 1.55; 10 ng/ml, fold change = 1.88; 30 ng/ml, fold change = 2.33) (Fig. 3A). Moreover, the upregulated *LGALS1* mRNA expression was suppressed by pretreatment with anti-IL1R1 (IL-1 receptor, type 1) neutralizing antibody (fold change = 1.37) compared to normal IgG treatment (fold change = 1.72) (Fig. 3B), suggesting a significant contribution of the IL-1β-IL1R1 axis to glial *LGALS1* expression. Further, to determine its downstream intracellular signaling, we employed specific inhibitors for extracellular signal-regulated kinase (ERK)1/2 (U0126), phosphatidylinositol-3 kinase (PI3K, LY294002), nuclear factor-κB (NF-κB, JSH-23), c-Jun N-terminal kinase (JNK) (SP600125), and p38 mitogen-activated protein kinase (MAPK) (SB203580). IL-1β-induced *LGALS1* mRNA levels were significantly reversed by ERK1/2 or PI3K inhibition (U0126, fold change = 1.27; LY294002, fold change = 1.14), but in contrast significantly augmented by p38 MAPK inhibition (SB203580, fold change = 2.19) (Fig. 3C). Treatment with SB203580 alone also increased *LGALS1* expression (Supplementary Fig. S2), suggesting the regulation of *LGALS1* transcription negatively via p38 MAPK and positively via ERK1/2 and PI3K in Müller glial cells. Additionally, we confirmed the impact of ERK1/2 or PI3K inhibition in protein levels as well (Fig. 3D).

Next, to confirm the *in vitro* IL1R1-mediated regulation of *LGALS1* mRNA (Fig. 3B), we performed immunofluorescence analyses to examine the existence of IL1R1 in human samples surgically collected from eyes with PDR. Müller glial cells with glial fibrillary acidic protein (GFAP)-positive signals expressed galectin-1 in fibrovascular tissues^15^. IL1R1 immunoreactivity was strongly positive in the fibrovascular tissue and co-localized with GFAP (Fig. 3E–G) and galectin-1 (Fig. 3H–J) signals, suggesting the production of galectin-1 protein via IL1R1 signaling in glial cells migrating into the proliferative tissue.

### AGE-Induced Expression of IL-1β in Macrophages via TLR4 Signaling

**Figure 4:**
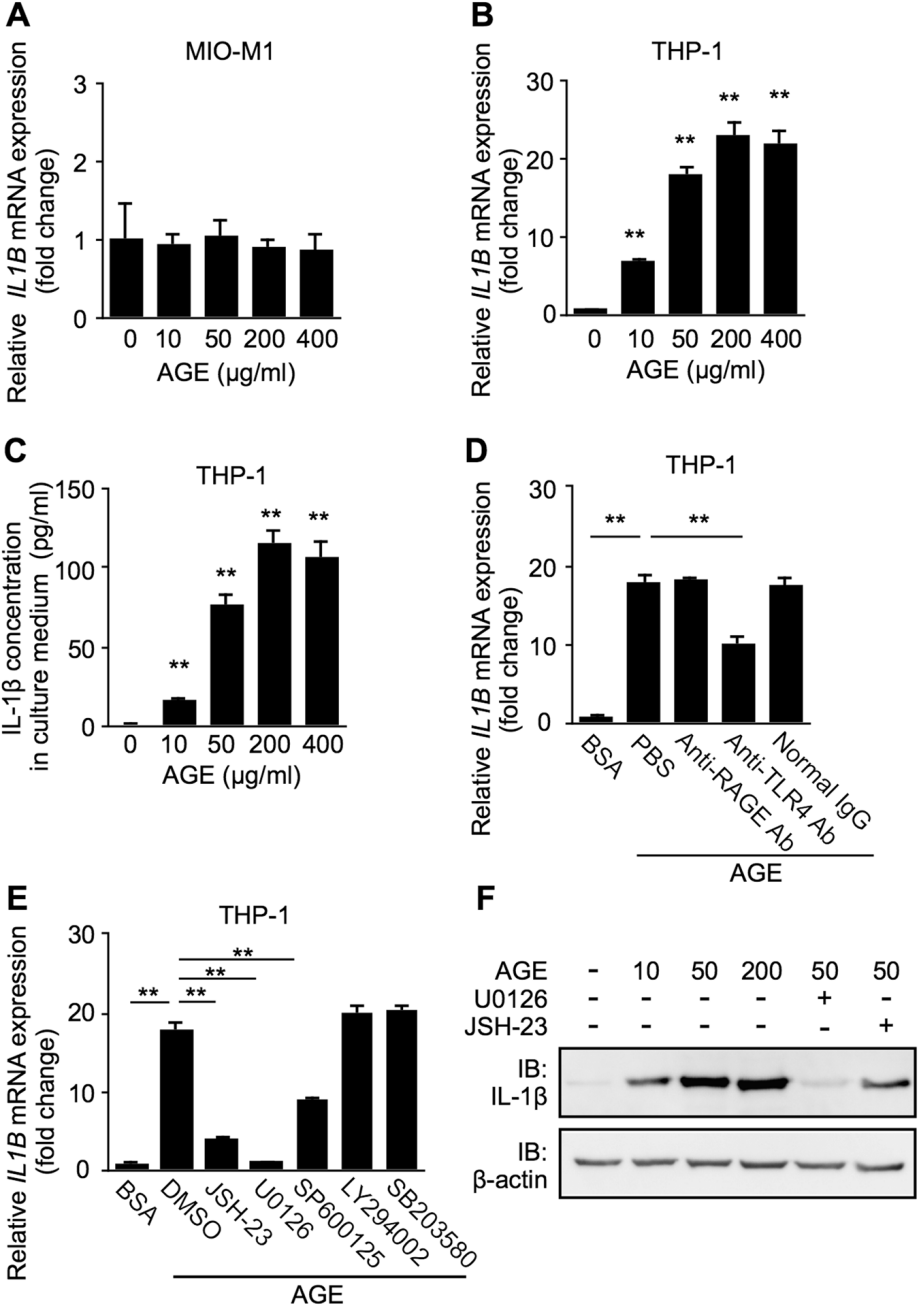
AGE-induced expression of IL-1β in macrophages via TLR4 signaling. (**A**) MIO-M1 was treated with AGE-BSA (10–400 μg/ml) for 24 hours, and *IL1B* expression levels were analyzed. (**B**,**C**) THP-1 was treated with AGE-BSA (10–400 μg/ml) for 24 hours, and *IL1B* gene expression levels in cell lysates (**B**) and IL-1β protein levels in culture media (**C**) were analyzed. (**D**) THP-1 was pretreated with neutralizing antibodies against RAGE (10 μg/ml) and TLR4 (10 μg/ml), in comparison with control normal IgG (10 μg/ml) for 30 minutes followed by treatment with AGE-BSA (50 μg/ml) for 24 hours, and *IL1B* gene expression levels were analyzed. (**E**,**F**) THP-1 was pretreated with each inhibitor at 10 μM 30 minutes before application with AGE-BSA (50 μg/ml) for 24 hours, and *IL1B* mRNA (**E**) and protein (**F**) expression levels were analyzed. **p < 0.01 (n = 8 per group). Full-length blots are presented in Supplementary Fig. S5.

Among various biological pathways involved in the pathogenesis of DR, AGE production has been regarded as one of the main contributors for microvascular complications in diabetes^8,9^. AGE levels were shown to increase in the vitreous fluid as well as in the serum of patients with DR^21^. Thus we hypothesized AGE as a potential inducer for IL-1β, which was shown to increase in eyes with DR but not non-diabetic retinal vascular occlusions^3–7^, so as to examine its cellular source in response to AGE-BSA. *IL1B* mRNA levels were not changed after AGE application to MIO-M1 or HRMEC (Fig. 4A, Supplementary Fig. S4), but in stark contrast, THP-1 clearly demonstrated a dose-dependent responsiveness to AGE in *IL1B* transcripts (10 μg/ml, fold change = 6.95; 50 μg/ml, fold change = 18.04; 200 μg/ml, fold change = 22.94; 400 μg/ml, fold change = 21.93) (Fig. 4B) and products in culture media (0 μg/ml = 1.49 pg/ml; 10 μg/ml = 16.64 pg/ml; 50 μg/ml = 77.75 pg/ml; 200 μg/ml = 115.69 pg/ml; 400 μg/ml = 107.36 pg/ml) (Fig. 4C).

AGE has been reported to bind with RAGE (receptor for advanced glycation endproducts) and TLR (toll-like receptor) 4 to activate inflammatory responses in chondrocytes and podocytes^22,23^. To identify signaling pathways involved in AGE-induced IL-1β production in human macrophages, we carried out blocking experiments. AGE-stimulated *IL1B* expression was significantly suppressed by pretreatment with anti-TLR4 neutralizing antibody (fold change = 10.01), but not with either anti-RAGE neutralizing antibody (fold change = 18.25) or normal IgG (fold change = 17.64) (Fig. 4D). We also confirmed that another RAGE inhibition using the RAGE antagonist peptide, RAP (ELKVLMEKEL), did not change *IL1B* expression (data not shown). MAPK and NF-κB signaling pathways have proven to be involved in AGE-induced inflammatory responses^24^. Pretreatment with JSH-23, U0126 and SP600125 effectively blocked AGE-induced *IL1B* expression (JSH-23, fold change = 4.12; U0126, fold change = 1.08; SP600125, fold change = 8.92) (Fig. 4E), suggesting the involvement of NF-κB, ERK1/2 and JNK signaling pathways in IL-1β-expressing macrophages. We also confirmed the impact of ERK1/2 or NF-κB inhibition on protein levels as well (Fig. 4F).

### AGE-Induced Expression of Galectin-1 via Cellular and Molecular Inflammatory Cues

**Figure 5:**
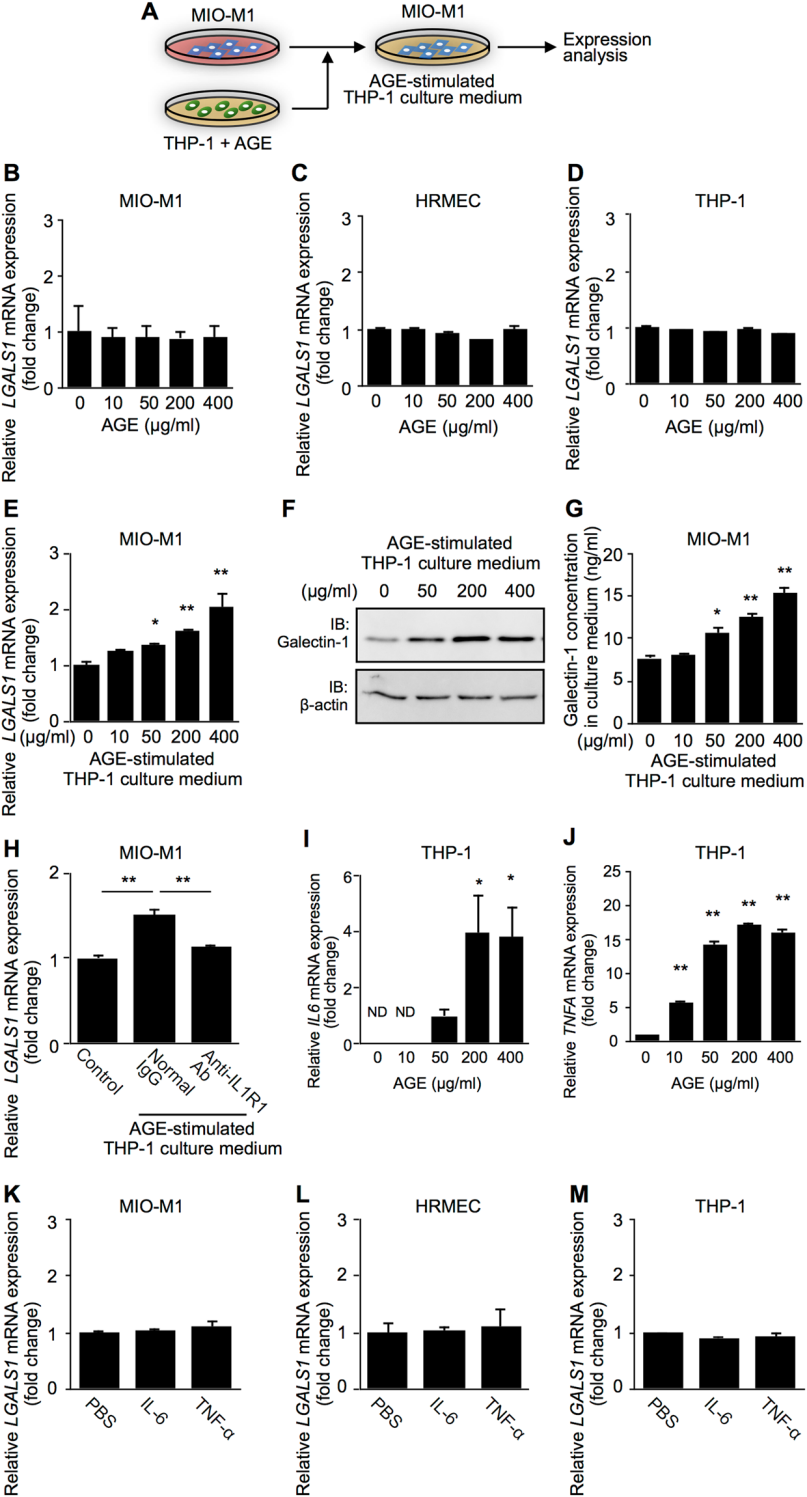
AGE-induced expression of galectin-1 via cellular and molecular inflammatory cues. (**A**) Experimental approach. (**B–D)** MIO-M1 (**B**) HRMEC (**C**) and THP-1 (**D**) were directly treated with AGE-BSA (10–400 μg/ml) for 24 hours, and processed for *LGALS1* gene expression. (**E–G**) MIO-M1 was treated with increasing concentrations of AGE-BSA-stimulated THP-1 culture medium for 48 hours, and processed for *LGALS1* gene (**E**) and protein ((**F**), cell lysates; (**G**), culture media) expression (n = 8 per group). Full-length blots are presented in Supplementary Fig. S6. (**H**) MIO-M1 was pretreated with a neutralizing antibody against IL1R1 (10 μg/ml) and control normal IgG (10 μg/ml) for 30 minutes, followed by treatment with AGE-BSA-stimulated THP-1 culture medium for 48 hours, and processed for *LGALS1* gene expression. (**I**,**J**) THP-1 was directly treated with AGE-BSA (10–400 μg/ml) for 24 hours, and processed for *IL6* (**I**) and *TNFA* (**J**) gene expression. ND, not detected. (**K–M**) MIO-M1 (**K**) HRMEC (**L**) and THP-1 (**M**) were treated with IL-6 (10 ng/ml) and TNF-α (10 ng/ml) for 24 hours, and *LGALS1* expression was analyzed. *p < 0.05, **p < 0.01 (n = 6 per group).

**Figure 6:**
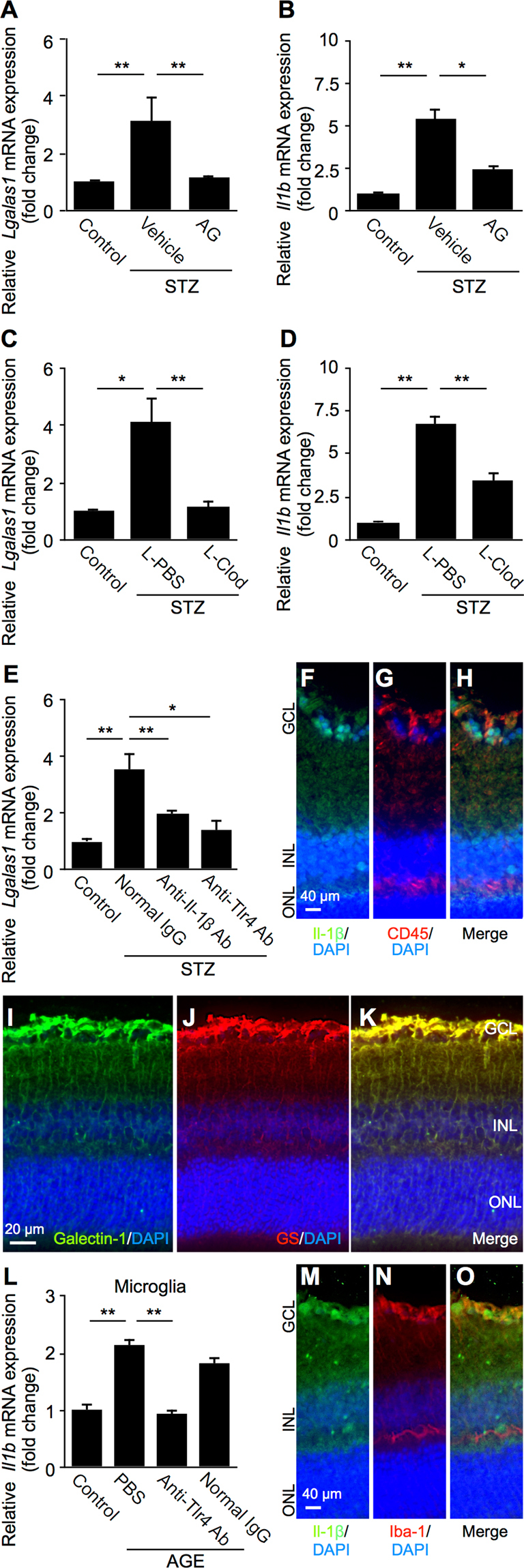
Diabetes-induced expression of retinal *Lgals1* via AGE-triggered inflammatory cues. (**A**,**B**) Retinal *Lgals1* and *Il1b* expression in mice with STZ-induced diabetes at 2 months. STZ mice received aminoguanidine (AG) for 7 consecutive weeks, and processed for gene expression. (**C**,**D**) Diabetic mice were given an intraperitoneal injection of 1-mg clodronate liposomes (L-Clod) 72 hours before *Lgals1* and *Il1b* gene expression analysis. Liposomes containing PBS were used as a vehicle control (L-PBS). (**E**) Neutralization antibodies against Il-1β (2.5 μg/eye) and Tlr4 (1.0 μg/eye) were injected intravitreally to STZ mice, in comparison with normal IgG (2.5 μg/eye), 48 hours before *Lgals1* gene expression analysis. (n = 4–6 per group). (**F–H**) Double labeling of Il-1β (*green*), CD45 (*red*) and DAPI (*blue*) in the retina of mice with STZ-induced diabetes at 2 months. Scale bar = 40 μm. (**I–K**) Double labeling of galectin-1 (*green*), GS (*red*) and DAPI (*blue*) in the diabetic retina. Scale bar = 20 μm. (**L**) Murine primary microglia was pretreated with a neutralizing antibody against Tlr4 (10 μg/ml), in comparison with PBS or control normal IgG (10 μg/ml) for 30 minutes followed by treatment with AGE-BSA (50 μg/ml) for 24 hours, and *Il1b* gene expression levels were analyzed. *p < 0.05, **p < 0.01 (n = 6 per group). (**M–O**) Double labeling of Il-1β (*green*), Iba-1 (*red*) and DAPI (*blue*) in the retina of mice with STZ-induced diabetes at 2 months. Scale bar = 40 μm.

To test a potential requirement of cellular (macrophage) and molecular (IL-1β) inflammatory cues in the DR-selective elevation of galectin-1 (Fig. 1A), we performed *in vitro* (Fig. 5) and *in vivo* (Fig. 6) experiments. *In vitro*, we investigated the effect of AGE-treated THP-1 culture supernatant on galectin-1/*LGALS1* expression in MIO-M1 (Fig. 5A), in order to recapitulate a pathological link between macrophages and Müller glial cells leading to galectin-1 production. Importantly, *LGALS1* mRNA expression was not altered following AGE administration directly to MIO-M1, HRMEC or THP-1 (Fig. 5B–D), while MIO-M1 applied with AGE-treated THP-1 culture supernatant led to a dose-dependent upregulation in *LGALS1* transcripts (10 μg/ml, fold change = 1.28; 50 μg/ml, fold change = 1.39; 200 μg/ml, fold change = 1.61; 400 μg/ml, fold change = 2.05) (Fig. 5E) and products in cell lysates (Fig. 5F), and galectin-1 secretion into culture media (0 μg/ml = 7.73 ng/ml; 10 μg/ml = 8.02 ng/ml; 50 μg/ml = 10.57 ng/ml; 200 μg/ml = 12.50 ng/ml; 400 μg/ml = 15.32 ng/ml) (Fig. 5G). Moreover, the upregulated *LGALS1* mRNA expression was suppressed by pretreatment with anti-IL1R1 neutralizing antibody (fold change = 1.14) compared to normal IgG treatment (fold change = 1.50) (Fig. 5H), suggesting the requirement of IL-1β secreted from AGE-stimulated macrophages to induce *LGALS1* expression in Müller glial cells.

Since AGE has been shown to induce various inflammatory responses in diabetes, we checked two representative cytokines IL-6 and tumor necrosis factor (TNF)-α, both of which are classically known to be associated with the pathogenesis of DR, in addition to IL-1β. AGE stimulation to THP-1 led to significant increases in mRNA expression levels of both *IL6* (0 μg/ml, not detected; 10 μg/ml, not detected; 50 μg/ml, fold change = 1.00; 200 μg/ml, fold change = 3.93; 400 μg/ml, fold change = 3.84) (Fig. 5I) and *TNFA* (10 μg/ml, fold change = 5.76; 50 μg/ml, fold change = 14.27; 200 μg/ml, fold change = 17.19; 400 μg/ml, fold change = 16.21) (Fig. 5J); however, *LGALS1* expression was not induced by either of the two cytokines in any of the cell lines MIO-M1, HRMEC or THP-1 (Fig. 5K–M), in consistence with the almost complete suppression of upregulated *LGALS1* mRNA levels achieved by anti-IL1R1 neutralizing antibody (Fig. 5H).

### Diabetes-Induced Expression of Retinal *Lgals1* via AGE-Triggered Inflammatory Cues

To further confirm whether the AGE-triggered inflammatory cues (Fig. 5H) are required for diabetes-induced retinal production of galectin-1 in mice (Fig. 1B), we performed the *in vivo* inhibition of AGE generation with aminoguanidine^25^. AGE accumulation in tissues including the retina was shown to be detected after 6 weeks of diabetes, and AGE inhibition attenuated pathological changes in mice at 8 weeks of diabetes^26–28^, leading us to fix the evaluation point of 2 months after STZ injection in the following experiments. *Lgals1* expression levels increased in the retina of mice with STZ-induced diabetes at 2 months compared to citrate buffer as a vehicle control (fold change = 3.16) (Fig. 6A), in accordance with the elevated protein concentration (Fig. 1B). Retinal *Il1b* expression levels also increased compared to a vehicle control (fold change = 5.39) (Fig. 6B), consistent with previous data on Il-1β production in diabetic rats^29^. Importantly, treatment with aminoguanidine (AG) led to significant suppression of both *Lgals1* (fold change = 1.19) and *Il1b* (fold change = 2.47) mRNA levels upregulated in the diabetic retina (Fig. 6A,B).

Next, in order to verify the requirement of IL-1β-secreting macrophages for glial production of galectin-1 (Fig. 5E–H), we assessed the effect of clodronate liposomes, which diminishes monocyte-derived macrophages^30,31^, on the retinal gene expression of *Lgals1* and *Il1b* in diabetic mice. Intraperitoneal application with clodronate liposomes (L-Clod) significantly downregulated retinal *Lgals1* (fold change = 1.16) and *Il1b* (fold change = 3.43) mRNA levels compared to treatment with PBS liposomes (L-PBS) (*Lgals1*, fold change = 4.16; *Il1b*, fold change = 6.77) (Fig. 6C,D).

In addition, we carried out antibody-based blockade of Il-1β and Tlr4 to validate the association of these macrophage-related molecules (Fig. 4D) with diabetes-induced *Lgals1* expression in the mouse retina. Intravitreal injections of anti-Il-1β and anti-Tlr4 neutralizing antibodies to animals with STZ-induced diabetes significantly reduced retinal *Lgals1* expression (anti-Il-1β, fold change = 1.96; anti-Tlr4, fold change = 1.41) compared to normal IgG treatment (fold change = 3.55) (Fig. 6E). These results suggested that AGE accumulation in diabetes initiates IL-1β-related inflammatory cues in macrophages followed by retinal galectin-1 upregulation.

To further investigate protein expression and tissue localization of Il-1β and galectin-1 in the retina of mice with STZ-induced diabetes, we performed double-staining immunofluorescence. Il-1β signal co-localized with CD45, a panleukocyte marker, around the ganglion cell layer (GCL) harboring the retinal vasculature (Fig. 6F–H), in consistence with the current data on *IL1B* and IL-1β expression in monocyte-derived macrophages (Fig. 4B–F). Galectin-1 immunoreactivity largely corresponded with the vertical (*i.e*., antero-posterior) extension of Müller cell processes throughout the entire retina, which were clearly stained with a Müller glial marker glutamine synthetase (GS) (Fig. 6I–K), supporting galectin-1/*LGALS1* expression in Müller cells (Figs 2 D, 3 A–D, 5E–H) and tissue localization of galectin-1 in IL1R1- and GFAP-positive activated glial cells (Fig. 3E–J). In agreement with changes in mRNA expression levels (Fig. 6A–E), both of Il-1β and galectin-1 protein signals were substantially diminished back to nearly normal levels after each treatment (data not shown).

In addition to monocyte-derived infiltrating macrophages, clodronate liposomes eliminate retinal and vitreal resident macrophages (*i.e*., microglia and hyalocytes, respectively) as well^31,32^, and microglial activation has also proven to be closely associated with diabetes-induced retinal inflammation^33,34^. Thus we investigated the possible involvement of microglia in the inhibitory effect of clodronate liposomes on diabetes-induced *Il1b* expression (Fig. 6D), using murine microglial primary cell culture stimulated with AGE-BSA. Importantly, *Il1b* mRNA levels significantly increased after AGE application to microglial cells (fold change = 2.13), which was almost completely inhibited by pretreatment with anti-Tlr4 neutralizing antibody (fold change = 0.93), but not with normal IgG (fold change = 1.84) (Fig. 6L). In the retina of mice with STZ-induced diabetes, Il-1β signal co-localized with ionized calcium-binding adapter molecule 1 (Iba-1), a microglial marker, mainly at the GCL (Fig. 6M–O), the site of predilection for retinal microglial cells activated by induction of diabetes to rats^35,36^.

### Tissue Co-localization of AGE, TLR4 and IL-1β in Macrophages

**Figure 7:**
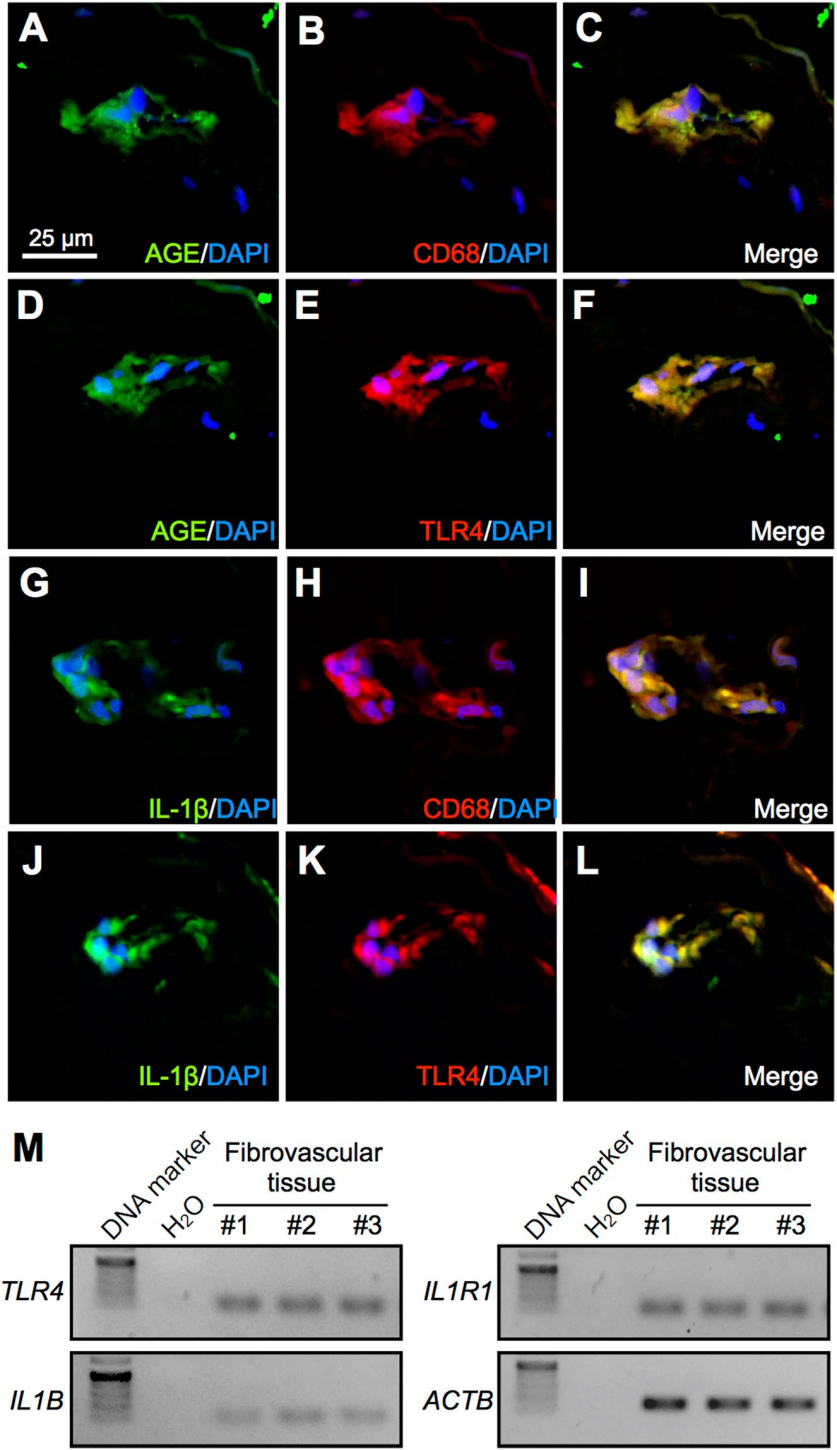
Tissue co-localization of AGE, TLR4 and IL-1β in macrophages. (**A–L**) Double labeling of AGE (*green*), CD68 (*red*) and DAPI (*blue*) (**A–C**); AGE (*green*), TLR4 (*red*) and DAPI (*blue*) (**D–F**); IL-1β (*green*), CD68 (*red*) and DAPI (*blue*) (**G–I**); IL-1β (*green*), TLR4 (*red*) and DAPI (*blue*) (**J–L**) in PDR fibrovascular tissues. Scale bar = 25 μm. (**M**) Gene expression of *TLR4*, *IL1B*, and *IL1R1* in PDR fibrovascular tissues. Full-length gels are presented in Supplementary Fig. S7.

To examine the tissue localization and expression of AGE, TLR4 and IL-1β, we carried out immunofluorescence for fibrovascular tissues surgically excised from human PDR eyes. Double-staining experiments demonstrated co-localization of AGE with CD68 (Fig. 7A–C), AGE with TLR4 (Fig. 7D–F), IL-1β with CD68 (Fig. 7G–I), and IL-1β with TLR4 (Fig. 7J–L) in the proliferative tissue, suggesting the AGE-induced production of IL-1β protein via TLR4 signaling in infiltrating macrophages, in concert with the current *in vitro* and *in vivo* data on TLR4-mediated *IL1B* expression in THP-1 (Fig. 4D) and AGE-induced *Il1b* expression in macrophages (Fig. 6B,D). We also confirmed tissue mRNA expression of *TLR4*, *IL1B* and *IL1R1* (Fig. 7M).

## Discussion

**Figure 8:**
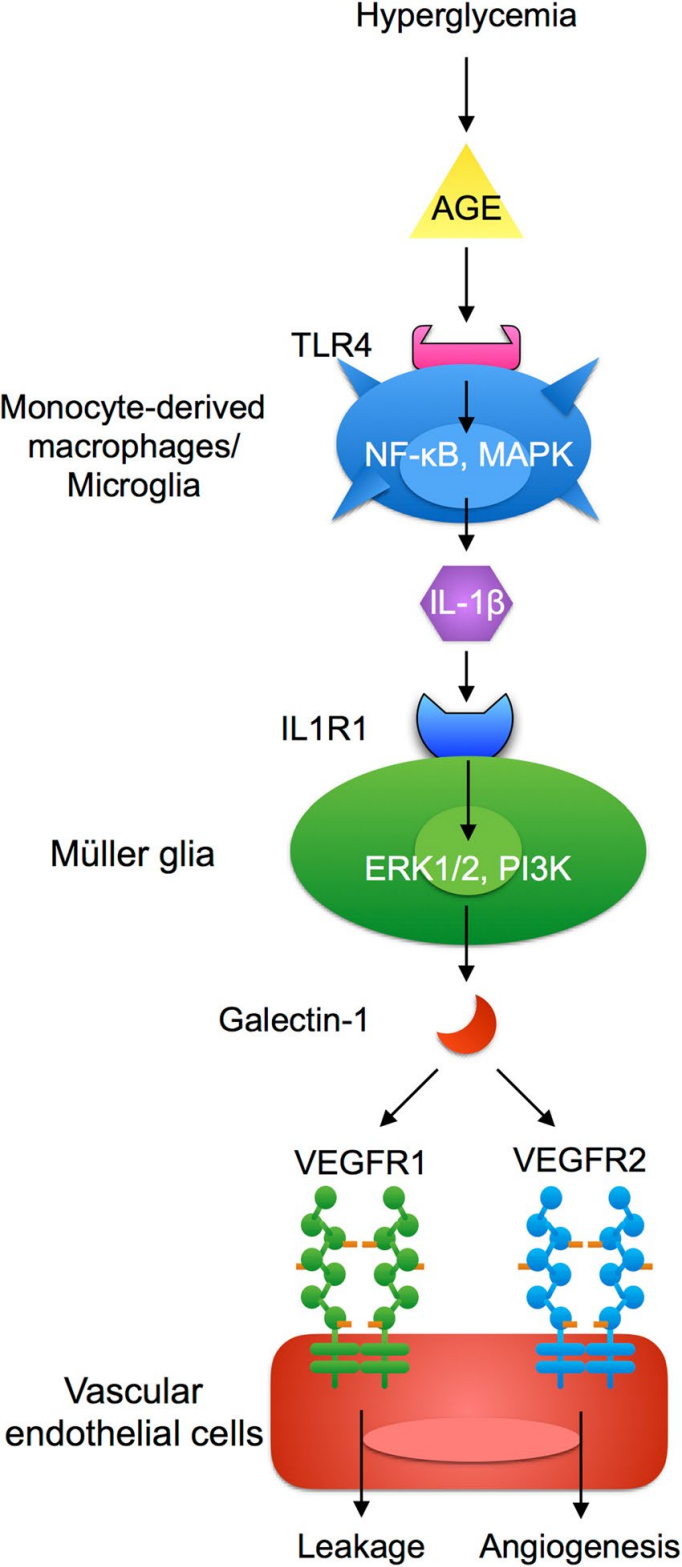
A schema showing the involvement of AGE-triggered inflammatory cues linking to galectin-1 upregulation in the pathogenesis of DR. AGE accumulation due to prolonged hyperglycemia triggers cellular (macrophage) and molecular (IL-1β) inflammatory cues linking to glial galectin-1 production, explaining the DR-selective elevation of galectin-1 along with disease activity (Fig. 1A). Galectin-1 binds to VEGFR1 and VEGFR2 in endothelial cells, leading to vascular leakage^16^ and angiogenesis^14,15^, respectively.

The present study revealed several important findings on the hypoxia-unrelated induction of galectin-1 selectively in the pathogenesis of DR but not non-diabetic retinopathies. Intracameral levels of galectin-1 protein were shown to increase in DR patients concurrently with the aggravation of clinical stages, but not in patients with other non-diabetic retinal vaso-occlusive diseases (Fig. 1). Administration with IL-1β to human retinal Müller glial cells enhanced galectin-1/*LGALS1* expression via IL1R1 together with intracellular signaling pathways ERK1/2 and PI3K (Figs 2, 3). AGE stimulation induced the expression of IL-1β through TLR4 activation and its downstream NF-κB, ERK1/2 and JNK signal transduction in human monocyte-derived macrophages (Fig. 4). Importantly, galectin-1/*LGALS1* expression was significantly elevated in Müller glial cells applied with AGE-stimulated macrophage culture supernatant, but not in Müller glial cells directly treated with AGE (Fig. 5). *In vivo* inhibition of AGE generation with aminoguanidine, depletion of macrophages with clodronate liposomes, and antibody-based blockade of Il-1β and Tlr4 significantly attenuated diabetes-induced retinal *Lgals1* expression in mice (Fig. 6). Induction of diabetes to mice facilitated retinal Il-1β production in Iba-1-positive resident macrophages (*i.e*., microglia), and AGE stimulation to murine microglial cells upregulated *Il-1b* expression via Tlr4 (Fig. 6). Immunofluorescence analyses showed co-localization of galectin-1 and IL1R1 in GFAP-positive glial cells (Fig. 3) and AGE, TLR4 and IL-1β in CD68-positive macrophages (Fig. 7) in fibrovascular tissues collected from PDR patients. These results suggest that diabetes-induced AGE accumulation activates cellular (macrophage) and molecular (IL-1β) inflammatory cues linking to glial galectin-1 production (Fig. 8), explaining the DR-selective elevation of galectin-1 along with disease activity beginning from the pre-ischemic stage of DME (Fig. 1).

During long-standing hyperglycemia in diabetes, excessive glucose forms covalent adducts with proteins gradually through a non-enzymatic process known as glycation. Increasing evidence has shown that hyperglycemia-induced AGE generation and subsequent activation of its receptors RAGE and TLR4 are involved in diabetic complications^24^, facilitating inflammatory and angiogenic processes^22,37–39^. In patients with DR, intraocular AGE accumulation has been suggested to contribute to disease onset and progression^21,40^. AGE-mediated stimulation of TLR4 was shown to induce signaling cascades such as ERK1/2 and NF-κB, triggering the release of pro-inflammatory cytokines and chemokines including IL-1β, IL-6, and TNF-α^22,41^. In concert with previous *in vivo* data showing the involvement of TLR4^42^ and IL-1β^43,44^ in diabetes-induced retinal inflammation and microvascular injury, our current *in vitro* and *in vivo* results as well as human data highlighted the significance of AGE-TLR4 signaling pathway to induce IL-1β in monocyte-derived macrophages and microglial cells (Figs 4, 6, 7), which was required for upregulation of galectin-1 in Müller glial cells (Figs 2, 3, 5). Although we and others already showed increased levels of galectin-1 in PDR^15^ and NVG^17^, this AGE-initiated inflammatory mechanism, which exists prior to the angiogenic stages of PDR and NVG, may explain the currently observed induction of galectin-1 in the earlier stage of DME (Fig. 1).

Recently, galectin-1 has been shown to recognize the *N*-glycans on VEGFR2, and regarded as a novel VEGFR2 ligand and endothelial cell mitogen^14,15^. Importantly, vitreous aspirates from PDR patients showed elevated levels of galectin-1 protein, which was not correlated with VEGF levels also elevated in the same individual samples, suggesting that these two molecules were independently regulated^15^. This is consistent with the current data showing the unelevated levels of galectin-1 in non-diabetic retinal vascular occlusions CRVO and BRVO (Fig. 1), both of which are governed by hypoxia-induced VEGF. Moreover, the present *in vitro* findings contrasted these two VEGFR2 ligands in terms of the preferential gene upregulation of *LGALS1* by IL-1β and *VEGF165* by hypoxia in Müller glial cells (Supplementary Fig. S1), which are activated in various retinal diseases. These findings emphasize that DR, another retinal vaso-occlusive disease, is a more complex clinical entity complicated by chronic and progressive microvascular abnormalities mediated by hyperglycemia-associated AGE in addition to hypoxia-induced VEGF.

Supporting our recent data showing the dissociation between VEGF and galectin-1 in PDR^15^, we confirmed the substantially different regulation of *VEGF165* mRNA expression in glial cells, vascular endothelial cells and macrophages treated with various DR-related stimuli (Supplementary Fig. S8) as compared with the current *LGALS1* data (Figs 2 and 5B–D). In anti-VEGF refractory tumors, galectin-1 secretion increased together with its enhanced binding to neovascular endothelial cells due to altered glycosylation patterns on VEGFR2 (*i.e*., decreased α2,6-linked sialic acid), leading to galectin-1-driven angiogenesis and tumor progression^14^. We have shown a significant increase in the total amount of *N*-glycans in PDR eyes, whereas α2,6-linked sialic acid was relatively decreased in glucose-stimulated human retinal vascular endothelial cells^45^. Based on these findings, the microenvironment of *N*-glycan profile in PDR eyes is suggested to facilitate the interaction of galectin-1 with VEGFR2, thus enhancing the function of galectin-1 as an angiogenic factor and elaborately escaping from anti-VEGF therapeutic efficacy.

This study has some limitations. The currently measured BRVO and CRVO samples were limited to those from eyes with macular edema but no retinal neovascularization. Although macular edema due to retinal vein occlusions appears to result mainly from ischemia-induced VEGF, it remains largely unclear why these retinal vaso-occlusive diseases failed to show elevated levels of ischemia-induced galectin-1. It is reasonable to speculate that the more advanced stages of CRVO and BRVO would exhibit higher levels of ischemia-induced galectin-1, which is also thought to contribute to the soaring rise in galectin-1 along with the severity of DR (Fig. 1A) on top of AGE-triggered galectin-1. Finally, the currently assessed cell types (*i.e*., Müller glia, vascular endothelial cells, monocyte-derived macrophages, and microglia) did not represent all the cellular participants in the pathogenesis of DR; therefore, we could not exclude the possibility of other cellular pathways leading to galectin-1 expression in the pathogenesis of DR.

In summary, diabetes-induced generation and accumulation of AGE were suggested to activate IL-1β-related inflammatory cues in monocyte-derived macrophages/microglia followed by Müller glia, linking to the upregulation of galectin-1 (Fig. 8) along with the severity of DR (Fig. 1). The present data underscore the inflammatory mechanism underlying the selective upregulation of galectin-1 in DR but not in non-diabetic, AGE-unrelated retinal vaso-occlusive diseases.

## Methods

### Reagents

The following antibodies were used: rabbit anti-galectin-1 (for human) and rabbit anti-AGE, Abcam (Cambrige, MA); mouse anti-CD68, mouse anti-TLR4 (for human), and rat anti-Tlr4 (for mouse), Affimetrix (Santa Clara, CA); mouse anti-GFAP, Leica (Exton, PA); rabbit anti-IL-1β (for human and mouse), Santa Cruz Biotechnology (Santa Cruz, CA); goat anti-Il-1β (for mouse), goat anti-IL1R1, mouse anti-RAGE, goat anti-galectin-1 (for mouse), and normal goat, mouse and rat IgGs, R&D Systems (Minneapolis, MN); rat anti-CD45, BD Biosciences (San Jose, CA); mouse anti-GS, Millipore (Temecula, CA); mouse anti-Iba-1, Wako Pure Chemical Industries (Osaka, Japan); rabbit anti-β-actin, Medical & Biological Laboratories (Nagoya, Japan); horseradish peroxidase-conjugated anti-rabbit IgG, Jackson ImmunoResearch Laboratories (West Grove, PA); AlexaFluor-conjugated anti-mouse, rabbit, rat and goat IgGs, Thermo Fisher Scientific (Waltham, MA). As for the anti-AGE antibody, both AGE-BSA and AGE-HSA (human serum albumin) are antigens immunized in rabbits to induce polyclonal antibodies, which the manufacturer confirmed can detect both AGE-BSA and AGE-HSA properly, showing minimal (<1%) cross reactivity with BSA and HSA. We further verified the specific binding of this antibody to AGE epitopes via immunoblot analysis (data not shown). Recombinant human IFN-γ, IGF-I, IL-12, IL-1β and IL-6 proteins were purchased from R&D Systems, and TNF-α from PeproTech (Rocky Hill, NJ). Aminoguanidine, BSA, dimethyl sulfoxide (DMSO) and STZ were from Sigma-Aldrich (St. Louis, MO). Polyvinylidene difluoride membrane, phorbol-12-myristate-13-acetate, and AGE-BSA were from Millipore. Endotoxin levels in the AGE-BSA were measured using an endotoxin testing kit (LAL Endotoxin Assay Kit, GenScript, Piscataway, NJ), and confirmed as negligibly low (<1 EU per 1 µg of protein) before use. Protease inhibitor cocktail tablets and 4′,6-diamidino-2-phenylindole (DAPI) were from Roche Applied Science (Indianapolis, IN). To block intracellular signaling pathways, the following inhibitors were used: JSH-23 for NF-κB, Sigma-Aldrich; SP600125 for JNK, Millipore; LY294002 for PI3K, Wako Pure Chemical Industries; SB203580 for p38 MAPK, Cell Signaling Technology (Danvers, MA); and U0126 for ERK 1/2, Promega (Madison, WI). Clodronate liposomes and PBS-containing liposomes were obtained from ClodronateLiposomes.com (Amsterdam, The Netherlands).

### Human Surgical Samples

For protein expression analysis of aqueous humor, a total of 50 patients were enrolled. Seven patients (3 males and 4 females, average age = 67.9 ± 3.2 years) were diagnosed with DME, 9 patients (5 males and 4 females, age = 62.2 ± 2.9 years) with PDR, 9 patients (6 males and 3 females, age = 64.9 ± 2.2 years) with NVG, 7 non-diabetic patients (2 males and 5 females, age = 70.4 ± 1.4 years) with macular edema due to BRVO, and 10 patients (6 males and 4 females, age = 66.8 ± 3.2 years) with macular edema due to CRVO. None of BRVO or CRVO patients developed retinal neovascularization. All the NVG eyes included in this study were at the advanced stage of DR, but not of BRVO or CRVO. Aqueous humor was collected just before the injection of anti-VEGF drugs. Control aqueous samples were collected by limbal paracentesis from 8 eyes of 8 age-matched, non-diabetic patients (5 males and 3 females, average age = 70.0 ± 2.6 years) undergoing routine surgery for age-related CAT. Undiluted aqueous humor samples were frozen rapidly and stored at −80 °C until further analyses. During surgery, 5 fibrovascular tissues were excised from PDR eyes and used for immunohistochemistry, and additional 3 fibrovascular tissues were processed for gene expression analyses. This study was conducted in accordance with the tenets of the Declaration of Helsinki and after receiving approval from the institutional review board of Hokkaido University Hospital. Written informed consent was obtained from all patients after an explanation of the purpose and procedures of this study.

### Cell Lines and Animals

The human Müller glial cell line (MIO-M1) was provided from Dr. G. Astrid Limb (UCL Institute of Ophthalmology, London, United Kingdom)^46^. The cells were cultured in DMEM containing 10% FBS (Thermo Fisher Scientific). Human monocytic cell line (THP-1) was obtained from Japanese Cancer Research Resources Bank (Osaka, Japan), grown in RPMI1640 media supplemented with 10% FBS, and differentiated into a macrophage-like phenotype after 48 hours with 200-nM phorbol-12-myristate-13-acetate. Human retinal microvascular endothelial cells (HRMEC) and CS-C medium optimized for HRMEC were purchased from Cell Systems (Kirkland, WA). Primary microglia isolated from C57BL/6 mouse brain and culture medium were purchased from Cosmo Bio (Tokyo, Japan).

C57BL/6 J mice were obtained from CLEA Japan (Tokyo, Japan). All animal experiments were conducted in accordance with the ARVO Statement for the Use of Animals in Ophthalmic and Vision Research, and approved by the Ethics Review Committee for Animal Experimentation of Hokkaido University. Procedures for murine model of STZ-induced diabetes were described in our previous report^15^. After 2 months, neutralizing antibodies for mouse Il-1β and Tlr4 were injected into the vitreous cavity. Clodronate liposomes were intraperitoneally applied. Aminoguanidine was dissolved in drinking water at a concentration of 1 g/l for 7 consecutive weeks soon after the establishment of diabetes (7 days after STZ injection) until the end of the study.

### ELISA

The protein levels of galectin-1 and IL-1β in human aqueous humors, mouse retina lysates, and cell culture supernatants were determined with human galectin-1 (R&D systems), mouse galectin-1 (Abcam), and human IL-1β (Affymetrix) ELISA kits per the manufacturers’ instructions. The optical density was determined using a microplate reader (Sunrise, TECAN, Männedorf, Switzerland).

### RT-PCR and Real-Time Quantitative PCR (qPCR)

Total RNA isolation and reverse transcription were performed from cells using SuperPrep Cell Lysis & RT Kit for qPCR (TOYOBO, Tokyo, Japan), and from tissues using PureLink RNA mini kit (Thermo Fisher Scientific) and GoScrip Reverse Transcriptase (Promega) following the manufacturers’ protocols. The following primers for genes were used: human *LGALS1* (galectin-1; forward 5′-CGC TAA GAG CTT CGT GCT GAA C-3′, reverse 5′-CAC ACC TCT GCA ACA CTT CCA G-3′), human *IL1B* (forward 5′-ACA GAT GAA GTG CTC CTTC CA-3′, reverse 5′-GTC GGA GAT TCG TAG CTG GAT-3′), human *IL1R1* (forward 5′-ATG AAA TTG ATG TTC GTC CCT GT-3′, reverse 5′-ACC ACG CAA TAG TAA TGT CCT G-3′), human *TLR4* (forward 5′-CGA GGA AGA GAA GAC ACC AGT-3′, reverse 5′-CAT CAT CCT CAC TGC TTC TGT-3′), human *VEGF165* (forward 5′-CAG ATT ATG CGG ATC AAA CCT CA-3′; reverse 5′-CAA GGC CCA CAG GGA TTT TC-3′), human *IL6* (forward 5′-CCA CTC ACC TCT TCA GAA CG-3′, reverse 5′-CAT CTT TGG AAG GTT CAG GTT G-3′), human *TNFA* (forward 5′-ACT TTG GAG TGA TCG GCC-3′, reverse 5′-GCT TGA GGG TTT GCT ACA AC-3′), human *ACTB* (β-actin; forward 5′-CTG GAA CGG TGA AGG TGA CA-3′, reverse 5′-AAG GGA CTT CCT GTA ACA ATG CA-3′), mouse *Lgals1* (forward 5′-GTC TCA GGA ATC TCT TCG CTT C-3′, reverse 5′-TCC CCG AAC TTT GAG ACATTC-3′, probe 5′-TTC AAT CAT GGC CTG TGG TCT GGT-3′), mouse *Il1b* (forward 5′-GCA ACT GTT CCT GAA CTC AAC T-3′, reverse 5′-ATC TTT TGG GGT CCG TCA ACT-3′), mouse *Gapdh* (glyceraldehyde-3-phosphate dehydrogenase; forward 5′-AGG TCG GTG TGA ACG GAT TTG-3′, reverse 5′-TGT AGA CCA TGT AGT TGA GGT CA-3′). Real-time qPCR was performed using the GoTaq qPCR Master mix (Promega), THUNDERBIRD Probe qPCR Mix (TOYOBO), and StepOne plus Systems (Thermo Fisher Scientific).

### Immunoblot Analyses

Cell extracts were lysed in SDS buffer and a protease inhibitor cocktail. After quantifying protein concentrations using BCA reagent (Thermo Fisher Scientific), proteins were resolved by 10% SDS-PAGE (polyacrylamide gel electrophoresis) and transferred to PVDF membrane by electroblotting. Membranes were blocked in TBS containing 5% skim milk, and probed with primary antibodies for galectin-1, IL-1β, and β-actin. Horseradish peroxidase-conjugated anti-rabbit IgG was used as a secondary antibody for chemoluminescence detection. Signal was obtained by enhanced chemoluminescence (Western Lightning Ultra, Perkin Elmer, Waltham, MA).

### Immunofluorescence Microscopy

Immunofluorescence analyses were performed as described previously^15,47^. Paraffin sections of fibrovascular tissues were deparaffinized and hydrated through exposure with xylene and graded alcohols followed by water. As a pretreatment, microwave-based antigen retrieval was performed in 10-mM citrate buffer (pH 6). Mouse eyeballs were fixed in 4% paraformaldehyde for 30 minutes on ice, incubated in PBS solution with increasing concentration of sucrose (10, 20, and 30%), and embedded in Frozen Section Compound (Leica, Exton, PA). Sections were probed with primary antibodies for galectin-1, AGE, CD45, CD68, GFAP, GS, Iba-1, IL-1β, IL1R1 and TLR4 followed by secondary antibodies. Nuclei were counterstained with DAPI, and sections were visualized under a Biorevo microscope (Keyence, Tokyo, Japan).

### Statistical Analyses

All the results are expressed as the mean ± SEM (standard error of the mean). Post-hoc test following the ANOVA was used for statistical comparison between groups. Differences between means were considered statistically significant when *p* values were <0.05.

## Electronic supplementary material

Supplementary Figures
