# Supplementary material for: Advanced glycation endproducts link inflammatory cues to upregulation of galectin-1 in diabetic retinopathy
Source: Sci Rep. 2017 Nov 23;7:16168. doi: 10.1038/s41598-017-16499-8 (PMC5700925; doi:10.1038/s41598-017-16499-8)
Supplement: Supplementary file 1 — Supplementary Figures [file 41598_2017_16499_MOESM1_ESM.pdf]

**Advanced glycation endproducts link inflammatory cues to upregulation of galectin-1 in diabetic retinopathy**

Atsuhiro Kanda, Yoko Dong, Kousuke Noda, Wataru Saito, and Susumu Ishida

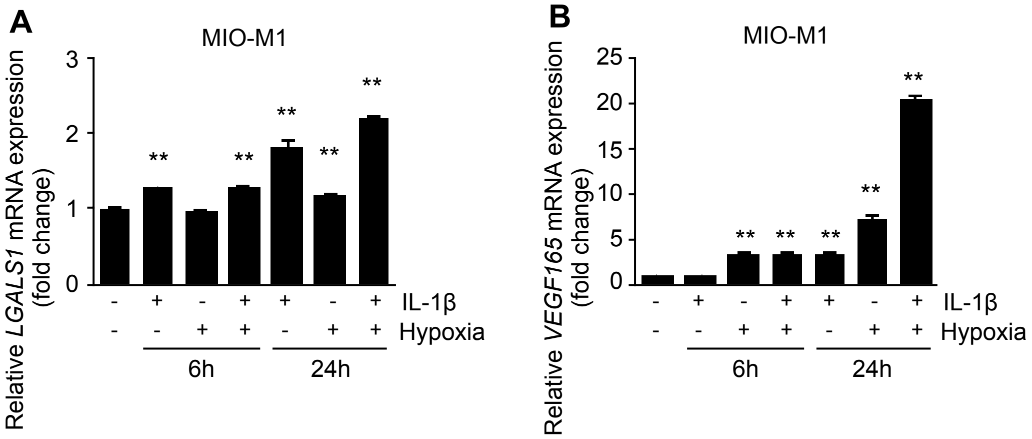


**Supplementary Figure S1. Preferential induction of *LGALS1* and *VEGF165* mRNAs by IL-1β and hypoxia, respectively**

**A, B,** MIO-M1 was stimulated with IL-1β (10 ng/ml) and/or hypoxia condition (1% $O_2$) for 6-24 hours, and *LGALS1* (**A**) and *VEGF165* (**B**) gene expression levels were analyzed. $^{**}p < 0.01$ (n = 6 per group).

**Advanced glycation endproducts link inflammatory cues to upregulation of galectin-1 in diabetic retinopathy**

Atsuhiro Kanda, Yoko Dong, Kousuke Noda, Wataru Saito, and Susumu Ishida

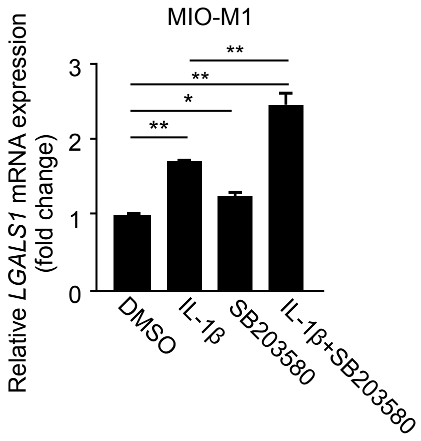


**Supplementary Figure S2.** ***LGALS1*** **expression in Müller glial cells via p38 MAPK**

MIO-M1 was pretreated with SB203580 (10 μM) for 30 minutes before stimulation with or without IL-1β (10 ng/ml) for 24 hours, and *LGALS1* gene expression levels were analyzed. $*p < 0.05$, $**p < 0.01$ (n = 8 per group).

**Advanced glycation endproducts link inflammatory cues to upregulation of galectin-1 in diabetic retinopathy**

Atsuhiro Kanda, Yoko Dong, Kousuke Noda, Wataru Saito, and Susumu Ishida

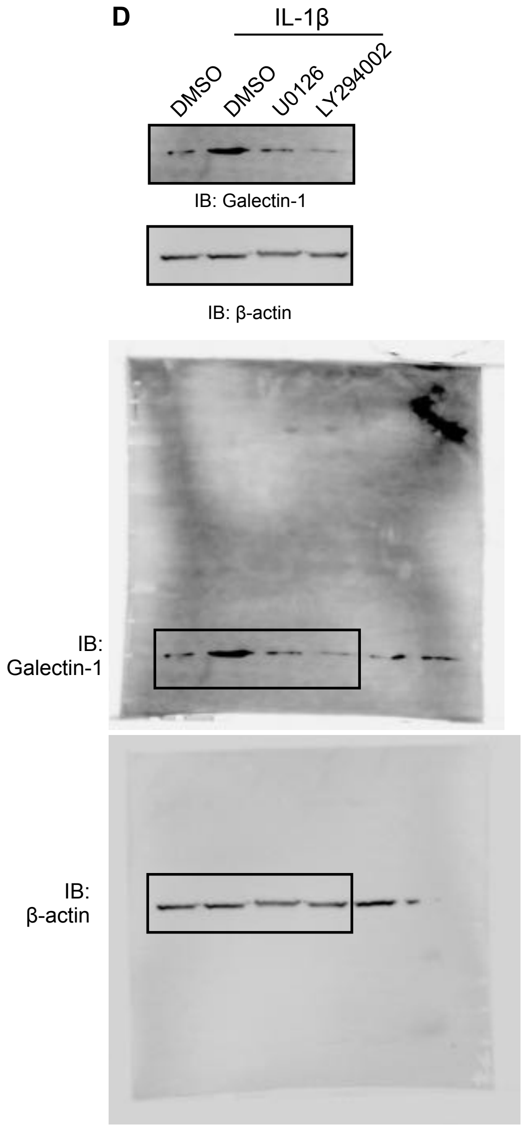


**Supplementary Figure S3. Full-length blots of Figure 3D**

**Advanced glycation endproducts link inflammatory cues to upregulation of galectin-1 in diabetic retinopathy**

Atsuhiro Kanda, Yoko Dong, Kousuke Noda, Wataru Saito, and Susumu Ishida

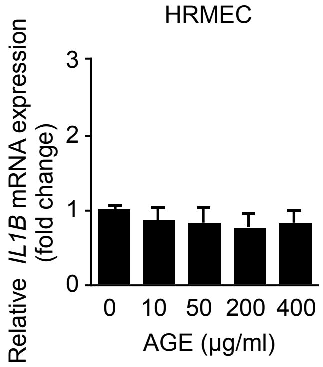


**Supplementary Figure S4. Unelevated expression of *IL1B* following AGE administration to retinal vascular endothelial cells**

HRMEC was treated with AGE-BSA (10-400 μg/ml) for 24 hours, and *IL1B* expression levels were analyzed. (n = 8 per group)

**Advanced glycation endproducts link inflammatory cues to upregulation of galectin-1 in diabetic retinopathy**

Atsuhiro Kanda, Yoko Dong, Kousuke Noda, Wataru Saito, and Susumu Ishida

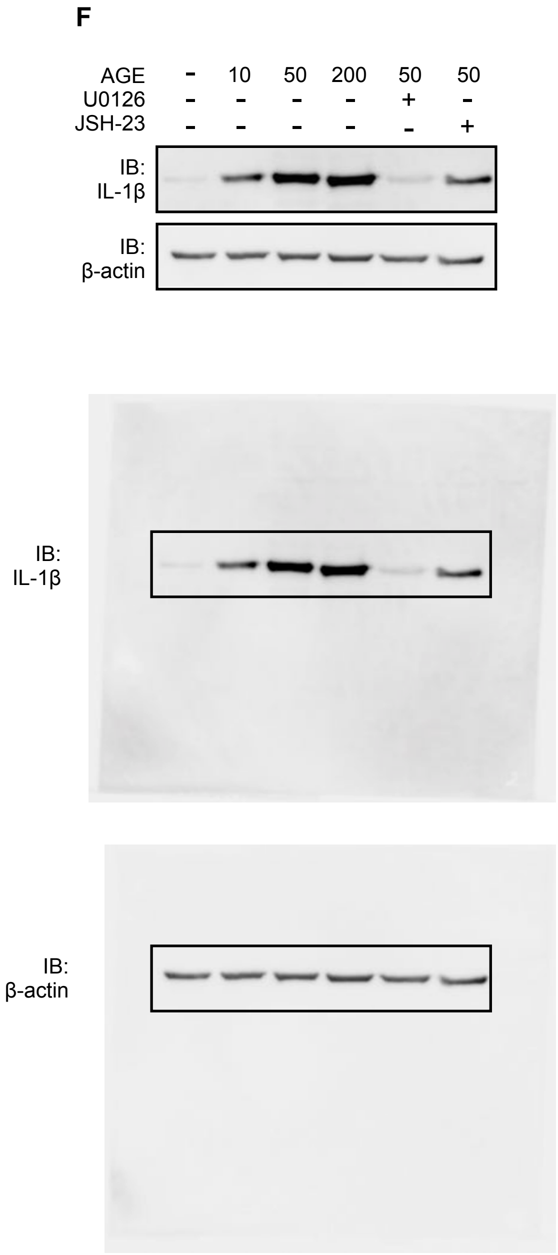


**Supplementary Figure S5. Full-length blots of Figure 4F**

**Advanced glycation endproducts link inflammatory cues to upregulation of galectin-1 in diabetic retinopathy**

Atsuhiro Kanda, Yoko Dong, Kousuke Noda, Wataru Saito, and Susumu Ishida

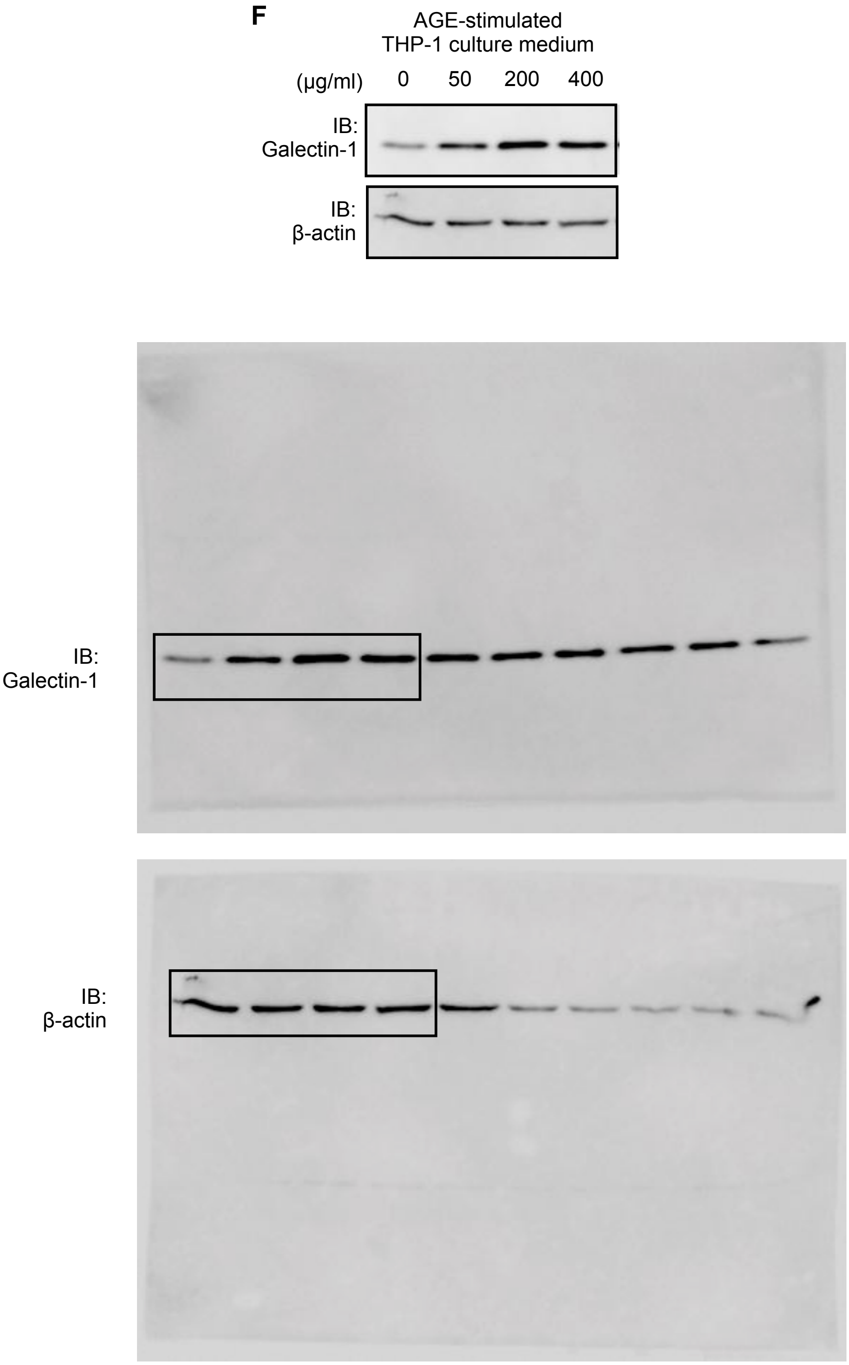


**Supplementary Figure S6. Full-length blots of Figure 5F**

**Advanced glycation endproducts link inflammatory cues to upregulation of galectin-1 in diabetic retinopathy**

Atsuhiro Kanda, Yoko Dong, Kousuke Noda, Wataru Saito, and Susumu Ishida

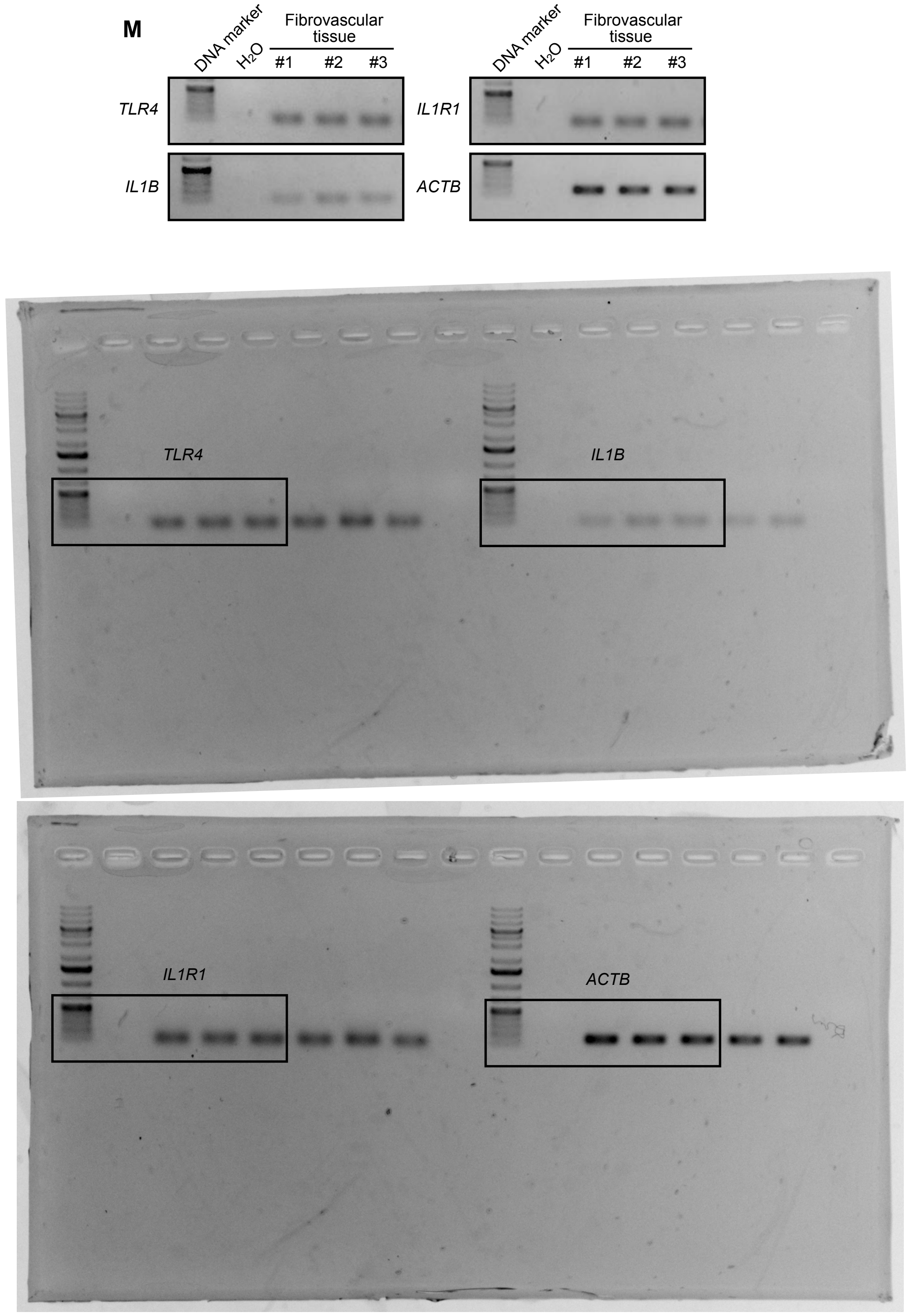


**Supplementary Figure S7. Full-length gels of Figure 7M**

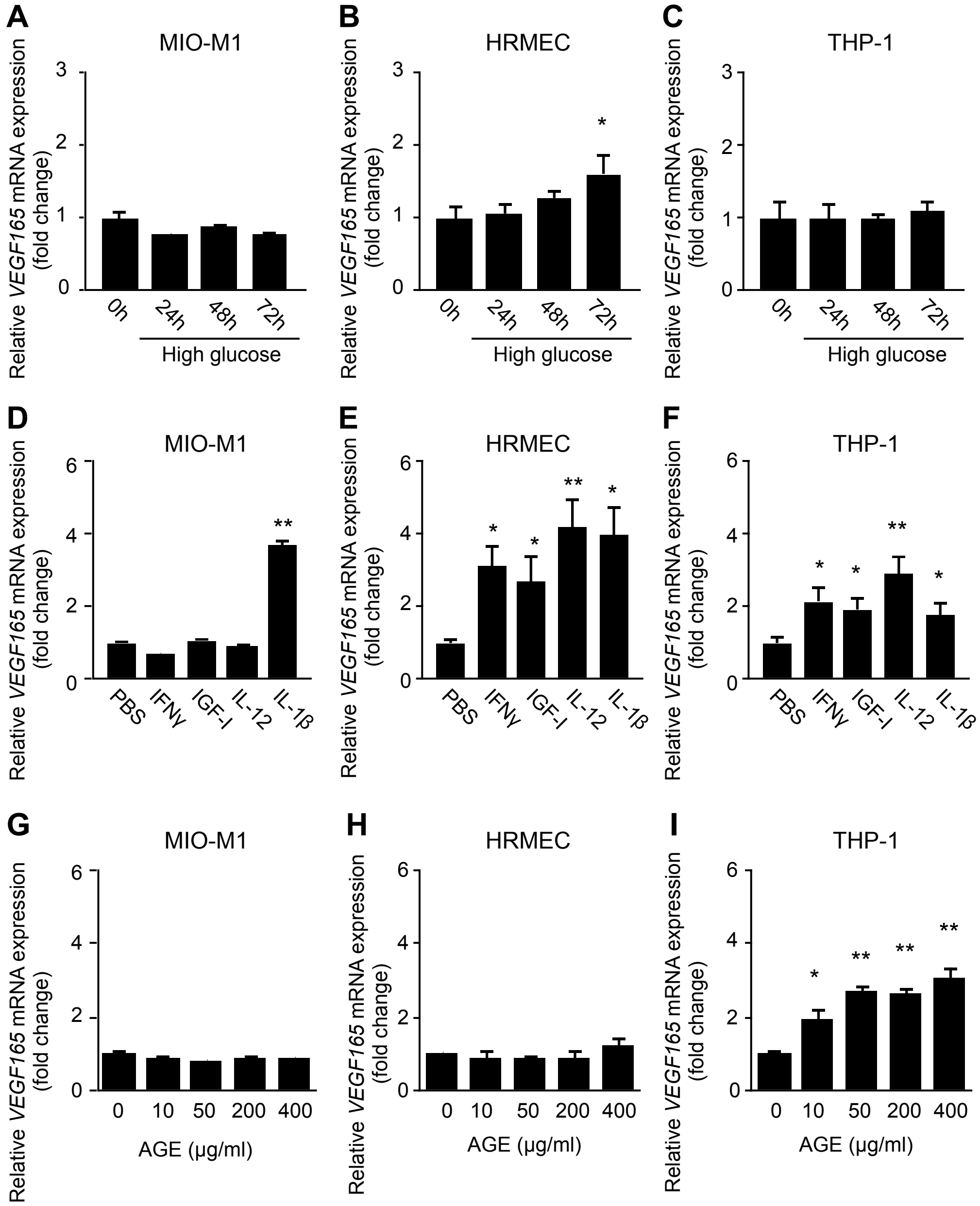


**Supplementary Figure S8.** ***VEGF165*** **mRNA expression following DR-related stimuli to Müller glial cells, retinal vascular endothelial cells and macrophages**

**A-C,** MIO-M1 (**A**), HRMEC (**B**) and THP-1 (**C**) were incubated with the medium containing 30-mM glucose for indicated time intervals, and *VEGF165* gene expression levels were analyzed. **D-F,** MIO-M1 (**D**), HRMEC (**E**) and THP-1 (**F**) were treated with IFN-γ (100 ng/ml), IGF-I (100 ng/ml), IL-12 (10 ng/ml), IL-1β (10 ng/ml) for 24 hours, and *VEGF165* expression levels were analyzed. **G-I,** MIO-M1 (**G**), HRMEC (**H**) and THP-1 (**I**) were treated with AGE-BSA (10-400 μg/ml) for 24 hours, and *VEGF165* expression levels were analyzed. $*p < 0.05$, $**p < 0.01$ (n = 8 per group).
